# Comparison of long-term results of carotid endarterectomy for asymptomatic carotid artery stenosis

**DOI:** 10.1007/s00772-018-0355-2

**Published:** 2018-02-05

**Authors:** S. Demirel, D. Böckler, M. Storck

**Affiliations:** 1 Department of Vascular and Endovascular Surgery, Department of Surgery, University Hospital Heidelberg, Im Neuenheimer Feld 110, 69120 Heidelberg, Germany; 2 Department of Vascular and Chest Surgery, Municipal Hospital Karlsruhe, Karlsruhe, Germany

**Keywords:** Asymptomatic stenosis, Internal carotid artery, Carotid endarterectomy, Carotid artery stenting, Evidence, Asymptomatische Stenose, Arteria carotis interna, Karotisendarterektomie, Karotisstent, Evidenz

## Abstract

This article summarizes the current study situation on treatment of asymptomatic carotid artery stenosis and discusses the evidence situation in the literature. The 10-year results of the ACST study have shown that in comparison to conservative treatment, carotid endarterectomy (CEA) has retained a positive long-term effect on the reduction of all forms of stroke. All multicenter randomized controlled trials comparing CEA with carotid artery stenting (CAS) and, in particular the SAPHIRE and CAVATAS studies, have in common that despite a basic evidence level of Ib, the case numbers of asymptomatic patients are too small for a conclusive therapy recommendation. In the overall assessment of the CREST study the resulting difference in the questionable endpoint of “perioperative myocardial infarction” in favor of the CAS methods, could not be confirmed for exclusively asymptomatic patients. In the long-term course of the CREST study, both methods were classified as equivalent, even when the 4‑year results of periprocedural and postprocedural stroke rates in the separate assessment of the asymptomatic study participants clearly favored the CEA. The results of the ACST-1 study showed an equivalent effect of both treatment methods with respect to all investigated endpoints; however, the unequal sizes of the groups in addition to the statistically insufficient case numbers put a question mark on the validity of the study results. The results of the ASCT-2 and CREST-2 studies are to be awaited, which also investigate the significance of “CEA versus CAS” (ASCT-2) and “CEA/CAS + best medical treatment (BMT) versus BMT alone” in only asymptomatic stenoses. The current S3 guidelines allow operative therapy to be considered in patients with a 60–99% asymptomatic carotid artery stenosis, because the risk of stroke is statistically significantly reduced.

## Zusammenfassung

Der folgende Artikel fasst die aktuelle Studienlage zur Therapie der symptomfreien Karotisstenose zusammen und diskutiert deren Evidenzlage in der Literatur. Die 10-Jahres-Ergebnisse der ACST-1-Studie haben gezeigt, dass die Karotisendaretrektomie (CEA) im Vergleich zur konservativen Therapie ihren positiven Langzeiteffekt in der Reduktion jedweder Schlaganfälle beibehalten hat. Alle multizentrisch randomisiert kontrollierten Studien mit dem Vergleich CEA versus Stent (*CAS* „carotid artery stenting“) und insb. die SAPHIRE- und CAVATAS-Studien haben gemeinsam, dass trotz eines grundsätzlichen Evidenzlevels Ib die Fallzahlen der symptomfreien Patienten für eine stabile Therapieempfehlung zu klein sind. Der in der Gesamtauswertung der CREST-Studie resultierende Unterschied des fraglichen Endpunkts „perioperativer Myokardinfarkt“ zugunsten der CAS-Methode konnte für die ausschließlich symptomfreien Patienten nicht bestätigt werden. Im Langzeitverlauf der CREST-Studie werden beide Methoden als gleichwertig eingestuft, auch wenn die 4‑Jahres-Ergebnisse der peri- und postprozeduralen Schlaganfallraten in der separaten Auswertung der symptomfreien Studienteilnehmer eine klare Favorisierung der CEA zulassen. Die Ergebnisse der ACT-1-Studie zeigen einen gleichwertigen Effekt beider Behandlungsmethoden hinsichtlich aller untersuchten Endpunkte. Die ungleiche Gruppengröße lässt jedoch neben der insgesamt statistisch nicht ausreichenden Fallzahl die Aussagefähigkeit dieser Studie hinterfragen. Abzuwarten sind die Ergebnisse der ACST-2- und CREST-2-Studien, die ebenfalls den Stellenwert der „CEA versus CAS“ (ACST-2) sowie „CEA/CAS + BMT (Best Medical Treatment) versus BMT als Single-Therapie“ bei nur symptomfreien Stenosen untersuchen. Die aktuelle S3-Leitlinie lässt bei Patienten mit einer 60–99 %igen symptomfreien Karotisstenose die operative Therapie in Erwägung ziehen, da das Schlaganfallrisiko statistisch signifikant reduziert wird.

## Introduction

Asymptomatic stenosis of the extracranial internal carotid artery (ICA) is a frequent incidental finding requiring further diagnostic investigation and treatment. Carotid stenosis is essentially an expression of generalized arteriosclerosis and, thus, also of systemic disease requiring adjunctive drug treatment/prevention with acetylsalicylic acid (ASA) and statins [1].

A number of studies have been published supporting only best medical treatment (BMT) compared with carotid endarterectomy (CEA) as the treatment of choice for primary stroke prevention [2]. On the other hand, according to results from randomized controlled studies (RCT) under the prerequisite of a perioperative stroke and mortality rate of <3%, CEA can be deemed more effective [3].

The evidence on preventive CEA for asymptomatic 60%–99% (North American Symptomatic Carotid Endarterectomy Trial, NASCET [4]) stenosis corresponds to level B and is thus not equivalent to symptomatic stenosis (level A).The S3 guidelines on the diagnosis, treatment, and follow-up of extracranial carotid stenosis states that CEA should be considered in patients with asymptomatic 60%–99% carotid stenosis, since it statistically significantly reduces, albeit it slightly, the risk of stroke in these patients [4]. This recommendation is based on results from the ACST-1 [5] and ACAS [6] studies, the data from which can only be partially extrapolated to the present day due to the evolving developments in drug therapy. On the other hand, there is a lack of completed randomized controlled studies investigating the value of the treatment methods, e.g. CEA vs. carotid artery stenting (CAS) vs. BMT in only asymptomatic carotid artery stenosis. Recruitment to the SPACE 2 study was stopped early before reaching the required number of study participants [7]. The patients that have already been recruited will nevertheless be evaluated. Thus, the preventive long-term effect (mean follow-up period of >2 years) of carotid reconstruction in asymptomatic carotid stenosis is of great importance. This review article presents the evidence on long-term CEA results in asymptomatic extracranial carotid stenosis.

## Material and methods

Randomized studies in PubMED (Medline) dealing with the methodological comparison of long-term outcome (>2-year follow-up) in the treatment of asymptomatic stenosis of the extracranial ICA between 1995 and 2016 were evaluated.

### 10-Year results of the ACST-1 study

**Fig. 1:**
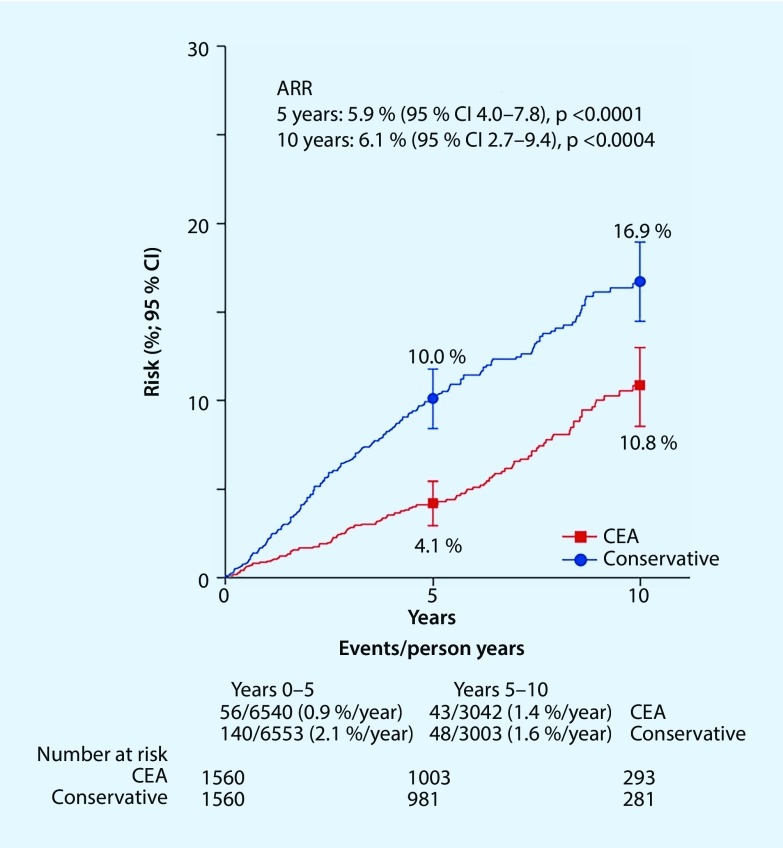
The 10-year risk for any periprocedural stoke: ACST-1 long-term results [7]. *ARR* absolute risk reduction, *CEA* carotid endarterectomy, *CI* confidence interval

Between 1993 and 2003, a total of 3120 patients with asymptomatic >60% extracranial internal carotid artery stenosis were included in a surgical and a conservative arm of the multicenter randomized controlled ACST-1 trial [8]. The percentage of asymptomatic patients undergoing surgery 5 years after randomization was 92.1% in the surgical arm and 16.5% in the conservative arm, and 92.2% vs. 23.5%, respectively, after 10 years (intention-to-treat analysis). There was no significant difference in the percentage of patients under the best possible adjuvant drug therapy either at the time of randomization (antihypertensive drugs: 51% vs. 55%, platelet aggregation inhibitors: 91% vs. 88%, anticoagulants: 5% vs. 6%, statins: 11% vs. 7%) or in long-term 10-year follow-up (antihypertensive drugs: 87% vs. 89%, platelet aggregation inhibitors: 88% vs. 89%, anticoagulants: 11% vs. 11%, statins: 80% vs. 82%). Excluding periprocedural events and non-stroke-related mortality, the risk for any stroke was 4.1% in the surgical arm and 10.0% in the conservative arm at 5‑year follow-up of patients aged <75 years (absolute risk reduction, ARR 5.9%) and 10.8% vs. 16.9% at 10 years (ARR 6.1%) (Fig. 1). At a ratio of 0.54 (95% confidence interval CI 0.43–0.68, *P* < 0.0001) of non-periprocedural stroke rates in the surgical vs. conservative arm, CEA showed a 46% reduction in the incidence of all strokes in the long-term. The proportional reduction in disabling or fatal stroke was similar to the proportional reduction in all strokes. Of the strokes for which the affected hemisphere was known, the greatest ARR was observed on the ipsilateral side (38 vs. 92 events, stroke rate ratio 0.43 (95% CI 0.28–0.68); *P* < 0.0001). Subgroup analysis of those patients with and without statins revealed a significant effectiveness of CEA in stroke prevention both at 5 years (ARR 3.4%, 95% CI 1.5–5.2; *P* = 0.0005 and ARR 10.8%, 95% CI 6.6–15.1; *P* < 0.0001, respectively) and at 10 years (ARR 5.8%, 95% CI 2.1–9.6; *P* = 0.002 and ARR 6.2%, 95% CI 0.4–12.8; *P* = 0.07, respectively). It can be concluded from the results of the ACST study that low complication CEA (<3% perioperative stroke rate) promotes the positive effect of drug therapy in long-term stroke prevention; however, due to the evolving developments in drug therapy, these data can only be partially extrapolated to the present time.

### Long-term results of RCTs on CAS vs. CEA in asymptomatic carotid stenosis

Of the altogether nine RCTs [9–17] published to date comparing CAS vs. CEA, four [11, 12, 15, 16] included patients with symptomatic and asymptomatic carotid stenosis, while one [17] included asymptomatic patients only.

The SAPPHIRE study [11], which primarily included high-risk surgical patients, was the only multicenter study in which the majority of randomized patients were asymptomatic. With the exception of the CAVATAS study [12], stents were used in the endovascular group in all RCTs. The CAVATAS study [12] also used only percutaneous transluminal angioplasty (PTA) in the period prior to 1994 and after 1994, either stenting or PTA was favored at the discretion of the interventionist. With >2500 patients, the CREST study [15] published in 2010 is the largest RCT yet to compare both treatment methods in medium-risk patients. Brooks et al. [18] (Lexington I) enrolled only symptomatic patients in the first part of their study and only asymptomatic patients in the second part [19] (Lexington II). The two study populations were merged for the purposes of evaluating long-term results in a further analysis [16]. No protection devices were used either in the CAVATAS [12] study or by Brooks et al. [16, 18, 19]. The median follow-up time of these studies was between 2 and 10 years. Details of study characteristics and results relating to asymptomatic patient populations can be found in Table 1.

**Table 1:** Study characteristics and endpoints of randomized controlled trials in which CAS was compared with CEA in asymptomatic patients

Study characteristics	SAPPHIRE [20]			CAVATAS [21]			CREST [22]			Brooks et al. (Lexington II) [19]			ACT-1 [17]		
Year of publication	2008			2009			2016			2014			2016		
Recruitment period	2000–2002			1992–1997			2000–2008			1998–2002			2005–2013		
Number of study participants and percentage of symptomatic vs. asymptomatic patients	334 Symptomatic (28.7%) Asymptomatic (71.3%)			505 Symptomatic (90%) Asymptomatic (10%)			2502 Symptomatic (52.4%) Asymptomatic (47.6%)			189 Symptomatic (55%) Asymptomatic (45%)			1453 Symptomatic (0%) Asymptomatic (100%)		
Lost to follow-up CAS CEA Total	14.4% 29.9%			ns ns			2.6% 3.8%			9%			1-Year follow-up 5.5% 5.5%		4-Year follow-up 8.5% 10.3%
Follow-up (median)	3.0 years			5.0 years			7.4 years			10 years^b^			5 years^b^		
Protection device used	95.6%			0.0%			96.1%			0.0%			100%		
**Endpoints for asymptomatic patients**															
–	CAS	CEA	*P*-value	CAS	CEA	*P*-value	CAS	CEA	*P*-value	CAS	CEA	*P*-value	CAS	CEA	*P*-value
Any stroke within the first 30 days or ipsilateral stroke between 31 and 1080 days	10.3%	9.2%	0.80	–	–	–	–	–	–	–	–	–	–	–	–
Combined endpoint (death, myocardial infarction, or any stroke within the first 30 days or ipsilateral stroke between 31 and 1080 days)	21.4%	29.2%	0.27	–	–	–	–	–	–	–	–	–	–	–	–
Combined endpoint (any stroke, myocardial infarction, or periprocedural death or postprocedural ipsilateral stroke)	–	–	–	–	–	–	7.1%	7.0%	0.95	–	–	–	–	–	–
Any stroke and periprocedural death	–	–	–	–	–	–	6.1%	4.8%	0.41	–	–	–	–	–	–
Any stroke including the periprocedural period Only postprocedural	–	–	–	–	–	–	6.1% 3.8%^a^	4.8% 3.7%^a^	0.41 0.92	–	–	–	–	–	–
Any stroke after 48 months, including periprocedural period	–	–	–	–	–	–	–	–	–	0%^c^	0%^c^	–	–	–	–
Ipsilateral stroke and fatal and non-fatal myocardial infarction, including the periprocedural period	–	–	–	–	–	–	–	–	–	0.9%^d^	4.1%^d^	<0.0001	–	–	–
Primary combined endpoint (death, any stroke, or myocardial infarction within the first 30 days, or ipsilateral stroke after 1 year)	–	–	–	–	–	–	–	–	–	–	–	–	3.8%	3.4%	0.69
Periprocedural death or severe stroke	–	–	–	–	–	–	–	–	–	–	–	–	0.6%	0.6%	ns
Periprocedural non-severe stroke	–	–	–	–	–	–	–	–	–	–	–	–	2.4%	1.1%	0.20

^a^Excluding patients with periprocedural stroke, myocardial infarction, or death

^b^Results are given as the cumulative incidence after 12.5 years (Brooks et al. [19]) and after 5 years (Rosenfield et al. [17])

^c^Results from the primary study (published in 2004)

^d^Percentages have been extrapolated from Fig. 4 in the original publication by Brooks et al. 2014 [16]

*ns* not specified, *CAS* carotid artery stenting, *CEA* carotid endarterectomy

The individual studies, including their long-term results, are presented in chronological order by year of publication and critically examined.

### SAPPHIRE (long-term results 2008)

Of 334 randomized patients, *n* = 260 patients (77.8%) underwent long-term follow-up (completed 3‑years follow-up) [20]. At *n* = 117, the proportion of asymptomatic (degree of stenosis >80%) patients in the CAS group was comparable to that in the CEA group (*n* = 120). There was no significant difference in the primary combined endpoint defined as death, myocardial infarction, or any stroke within the first 30 days, or death or ipsilateral stroke between 31 and 1080 days (Table 1). In their discussion, the authors emphasize that due to the small number of cases, it is not possible to draw conclusions on the investigation of non-inferiority of the CAS method in asymptomatic carotid stenosis. The markedly lower life expectancy of their patient groups compared with other RCTs (survival at 3 years: CAS 80% vs. CEA 75.8%) reflects the high-risk profile of the study participants. Study participants needed to meet at least one of the following criteria in order to be included as a high-risk patient in the study:

- Clinically significant heart disease (heart failure, abnormal stress test, or pending cardiac surgery)
- Severe lung disease
- Contralateral carotid occlusion
- Contralateral recurrent laryngeal nerve paralysis
- Status following neck dissection or radiotherapy
- Recurrent stenosis and age >80 years

Thus, no significant statement could be made about the representative general population of patients with asymptomatic carotid stenosis, in particular due to the selection bias.

### CAVATAS (long-term results 2009)

The CAVATAS study [21] randomized 505 patients, 90% of which had been symptomatic in the preceding 6 months, into a stent study arm or a surgical study arm. The main criticism of this study is that 75% of all patients in the interventional group were treated without protection devices or stents, meaning that the data cannot be extrapolated to current daily practice. There are also no published data on the small number of asymptomatic patients (Table 1).

### CREST (4-year and 10-year results)

In May 2010, the CREST investigators reported that the primary combined endpoint of periprocedural stroke, myocardial infarction, or death, or ipsilateral stroke within the first 4 years following randomization did not differ in the overall evaluation of all study participants when comparing CAS (*n* = 1262) vs. CEA (*n* = 1240) (7.2% vs. 6.8%; hazard ratio (HR) with CAS: 1.11; 95% CI 0.81–1.51; *P* = 0.51) [15]. At *n* = 1190, the number of asymptomatic patients was higher than in all other RCTs. A separate analysis at 4‑year follow-up showed no significant difference for the asymptomatic patient group in all endpoints when comparing both treatment methods, even though the difference in favor of CEA in the endpoint all periprocedural strokes or postprocedural ipsilateral stroke was close to the significance threshold (Table 2).

**Table 2:** The primary endpoint and its individual components in 1181 asymptomatic patients in a comparison of the two treatment groups in the CREST study [15]

Endpoint		Periprocedural period					4-year period (including periprocedural period)			
	CAS	CEA	Absolute treatment effect of CAS vs. CEA (95% CI)	Hazard ratio for CAS vs. CEA (95% CI)	*P*-value	CAS	CEA	Absolute treatment effect of CAS vs. CEA (95% CI)	Hazard ratio for CAS vs. CEA (95% CI)	*P*-value
	Number of patients (%±SE)		Percentage points			Number of patients (%±SE)		Percentage points		
*Myocardial infarction*										
Asymptomatic patients	7 (1.2 ± 0.4)	13 (2.2 ± 0.6)	−1.0 (−2.5–0.4)	0.55 (0.22–1.38)	0.20	–	–	–	–	–
*Any periprocedural stroke or postprocedural ipsilateral stroke*										
Asymptomatic patients	15 (2.5 ± 0.6)	8 (1.4 ± 0.5)	1.2 (−0.4–2.7)	1.88 (0.79–4.42)	0.15	24 (4.5 ± 0.9)	13 (2.7 ± 0.8)	1.9 (−0.5–4.3)	1.86 (0.95–3.66)	0.07
*Any periprocedural stroke or death or postprocedural ipsilateral stroke*										
Asymptomatic patients	15 (2.5 ± 0.6)	8 (1.4 ± 0.5)	1.2 (−0.4–2.7)	1.88 (0.79–4.42)	0.15	24 (4.5 ± 0.9)	13 (2.7 ± 0.8)	1.9 (−0.5–4.3)	1.86 (0.95–3.66)	0.07
*Primary endpoint (periprocedural stroke, myocardial infarction, or death, or ipsilateral stroke)*										
Asymptomatic patients	21 (3.5 ± 0.8)	21 (3.6 ± 0.8)	0.0 (−2.2–2.1)	1.02 (0.55–1.86)	0.96	30 (5.6 ± 1.0)	26 (4.9 ± 1.0)	0.7 (−2.1–3.4)	1.17 (0.69–1.98)	0.56

*CAS* carotid artery stenting, *CEA* carotid endarterectomy, *CI* confidence interval

Paraskevas et al. [23] published an article in 2013 criticizing the methodology used in CREST. The authors cite a 2004 article [24] of Hobson et al. showing that even within less than 3.5 years from the start of the study (December 2000), two thirds of the CAS population (789 out of 1262 patients, 62.5%) were included in the study as symptomatic patients. Only after 2005, once the recruitment of symptomatic patients had been suspended, were asymptomatic patients added. As such, the actual percentage of asymptomatic patients in the CAS group should not exceed 37.5%. The 47.1% reported is thus questionable. A further point of criticism was the definition of myocardial infarction as the primary study endpoint, which was put on a level with the events stroke and death. The comparatively high rate of myocardial ischemia (2.3%) in the surgical arm is likely due to the routine determination of heart enzymes 6–8 h following surgery and the laboratory value-based definition of infarction (creatine kinase-MB or troponin-T levels elevated to at least twice the upper limit and electrocardiogram changes or symptoms consistent with this).

**Fig. 2:**
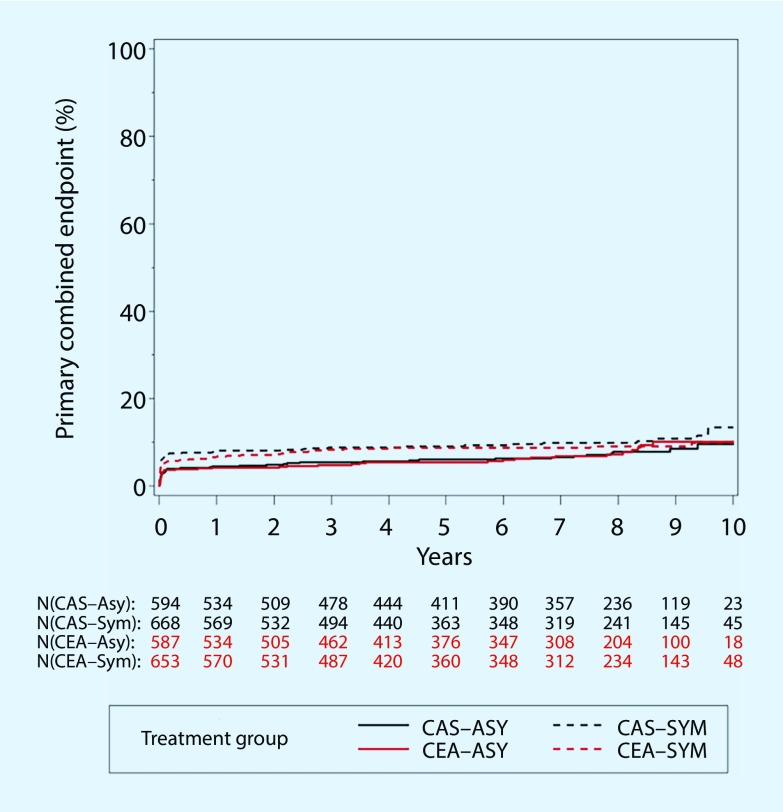
Primary combined endpoint any stroke, myocardial infarction, or death during the periprocedural period, or ipsilateral stroke within 10 years following randomization. Kaplan-Meier curves for asymptomatic (*ASY*) and symptomatic (*SYM*) patients in the carotid artery stenting (CAS) and carotid endarterectomy (CEA) treatment groups: CREST 10-year results [22]

Despite these criticisms, the significant difference in the overall evaluation of the periprocedural myocardial infarction rate in favor of the CAS procedure could not be confirmed in the analysis of only asymptomatic patients (Table 2). Much like the 4‑year results, there was no significant difference at 10-year follow-up in patients with asymptomatic carotid stenosis in terms of the primary combined endpoint ([22], Table 1; Fig. 2).

### Single-center RCT (Lexington II)

Between 1998 and 2002 Brooks et al. [19] randomized 85 high and medium-risk patients with asymptomatic carotid stenosis. All patients in the CAS group were treated without protection devices but with stents, in contrast to the CAVATAS study. No cerebral events were observed in either group both during the periprocedural period and at 4‑year follow-up (Table 1). Long-term results in the combined endpoint ipsilateral stroke, fatal and non-lethal myocardial infarction, including the first 30 postprocedural days showed cardiac events to be significantly more frequent in the CEA group (Table 1), while rates of ipsilateral stroke did not differ (*p* > 0.05). The authors saw a correlation between the significantly more frequent cardiac events and elevated cardiac enzymes in the periprocedural period in the CREST study CEA group, and therefore suggested a negative predictor that possibly manifests in increased cardiac morbidity and mortality in the long term; however, this interpretation is purely hypothetical, particularly since the authors of the Lexington II study did not determine or analyze any heart enzymes.

### ACT-1

**Fig. 3:**
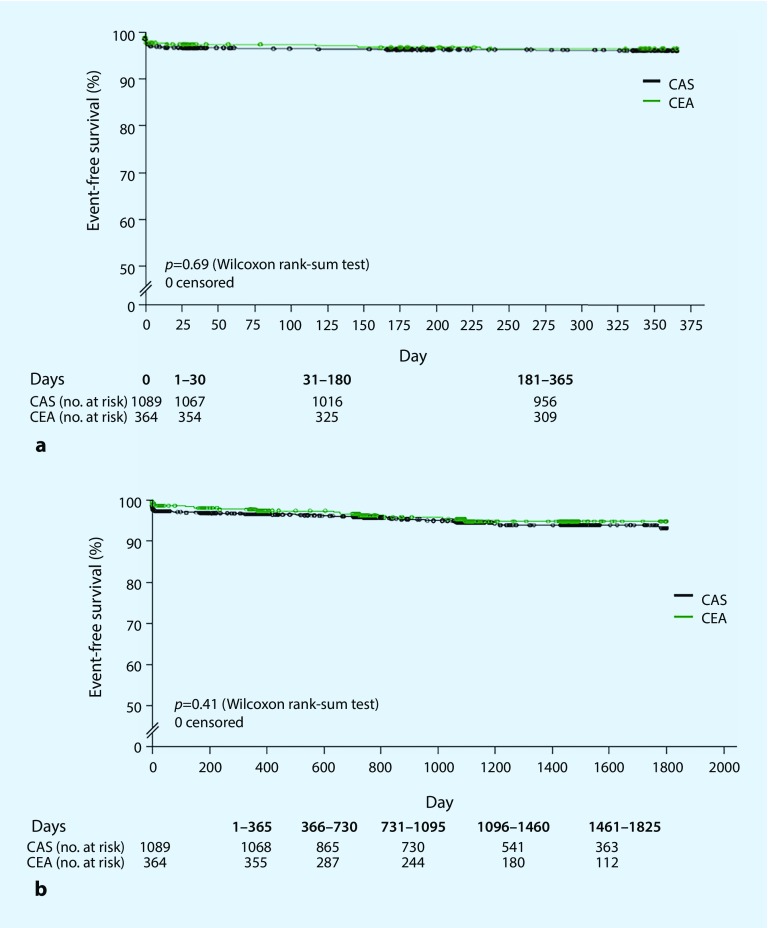
Kaplan-Meier curves for event-free survival in the ACT-1 study [17]. **a** Primary combined endpoint (death, any stroke, or myocardial infarction within the first 30 days, or ipsilateral stroke after 1 year). **b** Secondary endpoint (any stroke during follow-up of up to 5 years); *CAS* carotid artery stenting, *CEA* carotid endarterectomy

The ACT-1 study [17] is the only multicenter controlled study to date that has investigated the value of CAS vs. CEA in the treatment of only asymptomatic patients at low to moderate risk (<79 years of age). In total 1453 patients were included with remarkably disparate group sizes (CAS *n* = 1089; CEA *n* = 364). Similar to the CREST study, no differences were seen in terms of the primary combined endpoint death, any stroke, or myocardial infarction within the first 30 days, or ipsilateral stroke at 1 year, as shown by the almost identical Kaplan-Meier curves in Fig. 2. Neither group showed any difference in the stroke rate during the periprocedural period (Table 1) or in long-term follow-up (Fig. 3). In line with the separate analysis of asymptomatic patients in the CREST study, the ACT-1 study found no difference in periprocedural myocardial infarction rates between CEA and CAS in (0.5% vs. 0.9%; *P* = 0.41).

## Conclusion

- The treatment of patients with asymptomatic carotid stenosis continues to be approached differently. The superiority of CEA compared with drug therapy in patients with symptomatic stenosis is established, assuming surgery is performed at a risk of less than 6% (stroke rate/mortality). The benefit conferred by CEA particularly in the long term for asymptomatic patients is less well established. Surgery as an adjunct to drug therapy is slightly superior if performed at a stroke or mortality risk of less than 3%.
- CAS has been further developed as an alternative in recent years and is now used in asymptomatic patients despite a lack of convincing evidence. Substantial progress has also been made in the primary drug prevention of cerebrovascular and cardiovascular diseases.
- Since the large randomized studies on CEA in asymptomatic patients presented here were conducted more than 10 years ago, the patients in these studies were mostly not treated according to current prevention standards in terms of BMT. Adequately sized randomized studies yielding long-term results are not yet available.
- Due to overly slow recruitment and the difficulties associated with providing all three treatment options within the necessary quality requirements at all study centers, the SPACE 2 trial had to be discontinued.
- In accordance with the S3 guidelines, CEA is considered in 60%–99% asymptomatic stenosis, since the risk of stroke can be statistically significantly reduced, albeit slightly. A complication rate <3% is a prerequisite. The value of the three treatment approaches (CEA, CAS, and BMT) relative to each other still needs to be investigated in controlled three-arm studies.
- CAS can be considered as a possible alternative in existing indications if similar quality requirements as for CEA and complication rates <3% are met [4]. The results of the ACST-2 and CREST-2 studies, which are currently also investigating the value of CEA vs. CAS (ACST-2) and CEA/CAS+BMT vs. BMT as a single therapy in asymptomatic stenosis, are pending.
